# Drug‐Drug Interactions in Elderly Adults in Dentistry Care: a Cross-Sectional Study

**DOI:** 10.30476/DENTJODS.2021.91067.1549

**Published:** 2022-12

**Authors:** Elham Abbaszadeh, Niloofar Ganjalikhan Hakemi, Maryam Rad, Molook Torabi

**Affiliations:** 1 Dept. of Oral Medicine, School of Dentistry, Kerman University of Medical Sciences, Kerman, Iran; 2 Dentist, Private Practice, Kerman, Iran; 3 Oral and Dental Diseases Research Center and Kerman Social Determinants on Oral Health Research ‎Center, Kerman University of Medical Sciences, Kerman, Iran; 4 Dept. of Maxillofacial Pathology, Dental School, Kerman University of Medical Sciences, Kerman, Iran

**Keywords:** Drug interaction, Elderly, Dentistry

## Abstract

**Statement of the Problem:**

Given the increase in the population of the elderly patients and the risk of systemic diseases in these individuals, the prevalence of the intake of various drugs is higher in elderly patients, which exposes them to the side effects of drugs including potential drug-drug interactions (DDIs).

**Purpose:**

Therefore, the present study is an attempt to evaluate the drug interactions between the drugs used by the elderly patients visiting Kerman School of Dentistry and the common dental drugs in 2020.

**Materials and Method:**

This cross-sectional study was conducted on the elderly patients (≥60 years (who referred to Kerman School of Dentistry for dental problems. After obtaining the oral informed consent and collecting demographic information, the drugs used by the patients and their systemic diseases were questioned, listed, and compared with the drugs mentioned in their files. The drug interactions with the common dental drugs were studied in the elderly patients using the www.drugs.com website. Chi-square, T, ANOVA, Kruskal-Wallis, and Mann-Whitney tests were used to compare the variables. The significance level was 0.05.

**Results:**

Of participants included in this study, 78 (52%) were female and 72(48%) were male. The average age of these patients was 71.27 6.32 years. The most common systemic diseases were hypertension (57.3%), heart diseases (42.0%), and diabetes mellitus (40.7%). Our analysis of the DDIs between 11 commonly prescribed dental drugs and 95 drugs used by the patients revealed 212 DDIs (21.7% minor, 68.3% moderate, and 9.9% major interactions). There was a significant relationship between the number of drugs and DDIs, whereas DDIs had no significant relationship with gender and educational level.

**Conclusion:**

The results reflected the high percentage of DDIs among the patients. In addition, there was a significant relationship between polypharmacy, which is highly prevalent among the elderly patients, and drug interactions.

## Introduction

According to the definition by the World Health Organization, the old age refers to the age of 60 years and higher in the developing countries and the age of 65 years and higher in the developed countries [ 1 ]. From 2000 to 2050, the population over the age of 60 years old increases from 600 million to 2 billion people. These individuals mostly belong to the underdeveloped countries. In most underdeveloped countries, the population of people over the age of 60 years is growing faster than the other age groups. Iran’s population, especially the population over the age of 65 years, is also increasing drastically. Therefore, given the rapid growth of the aging process, the elderly population of Iran has increased dramatically over the past 40 years, and this trend is expected to be more than doubled in the next 20 years [ 2 ]. 

The aging process undermines one’s physical and mental ability, thereby increasing the prevalence of numerous diseases such as Alzheimer’s disease, Parkinson’s disease, vascular dementia, stroke, arthritis, osteoporosis, and fractures.

 Hence, most of these patients take many drugs for treatment of their different diseases [ 3 ]. Besides, approximately 70 to 90 percent of the elderly take at least one oral drug a day, while two to five drugs are prescribed every year for an elderly on average [ 4 ]. The potential for drug interactions increases with the number of drugs. The estimated risk of drug side effects is 13% for 2 drugs, 58% for 5 drugs, and 82% for 7 drugs or more [ 5 ]. Therefore, multidrug therapies are always linked to a high rate of potentially serious drug interactions and the adverse side effects of drugs [ 6 ].

The rate of incidence of all drug reactions in the elderly patients is at least twice the youth because both the physicians’ prescription models and the intake of drugs by the patients can be erroneous [ 3 ]. Drug-drug interactions (DDIs) occur when another factor (the second drug) affects the pharmacokinetics or pharmacodynamics of a characteristic drug (the first drug) and exacerbates a known adverse side effect of the characteristic drug (the first drug), which can increase the toxicity or reduce the therapeutic effect of the drug [ 7 ].

The prevalence of DDIs in the hospitalized elderly patients is reported to be approximately 10% [ 8 ]. Aging, comorbidity of chronic diseases, multiple comorbidities, polypharmacy, and the introduction of new drugs to the market are factors that contribute to the frequency and severity of DDIs [ 7 ].

In the elderly, two-thirds of the adverse drug side effects result from the intake of drugs such as corticosteroids, nonsteroidal anti-inflammatory drugs, cardiovascular drugs, and psychiatric drugs. Some of the most prevalent examples of these side effects include dizziness and cognitive disorders, gastrointestinal disorders such as bleeding and gastritis, syncope, extrapyramidal complications, and arrhythmia [ 9 ].

Drug interactions are classified into three classes:

1- Class I: Major

This category of interactions is substantially important. Reliable documents, evidence and reports are available on these interactions, and the risk occurs immediately within 24 hours with high intensity.

2- Class II: Moderate

These interactions have moderate clinical importance and more documents, evidence, and reports on these interactions are required. They also pose the risk of delayed attacks after 24 hours.

3- Class III: Minor

These interactions may occur yet they are clinically insignificant for some reasons including (1) there are very few documents, evidence, and documented reports on them, (2) they pose insignificant risks to the patients, and ( 3) the risk of interactions is considerably low [ 10 ].

Due to the increasing population of the elderly and their need for dental treatment and because these patients are treated with various drugs, it is necessary for dentists to be aware of drug interactions in these patients. Besides, the importance of prescription of drugs for the elderly in the dental treatments should be considered. Although DDIs in elderly patients has been studied in various fields of medicine, yet there is no study on the drugs prescribed in dentistry. Studying the drug interactions in the healthy population in Iran is one of the concerns that call for careful attention. Hence, this study was conducted to analyze the drug interactions between the common dental drugs with 95 drugs in 150 elderly patients. 

## Materials and Method

This descriptive cross-sectional study was conducted in 2020 at Kerman School of Dentistry on 150 elderly patients over the age of 60 years who visited this school of dentistry to receive medical dental treatments. Using the N= (p.q.z2)/d2 formula and a prevalence rate of 40 percent (according to the CDC report, 40 percent of the elderly need medical dental actions [ 11 ]) the resulting sample size was 150: *p*= 40%, q= 60%, Z=1.96, α=0.05, d=0.08

This project was approved by Kerman Medical University ethical committee (# IR.KMU. REC.1398.587).

After obtaining the oral informed consent of the patients, their demographic information including their age, gender, and education level were recorded based on their files. In addition, the patients were asked about the drugs they used and their systemic diseases and the information was compared to the drugs mentioned in the patients’ files. The drug interactions between these drugs and the common dental drugs were studied by a senior student in general dentistry that was trained accordingly.

The drug interactions were analyzed based on the www.drugs.com website. In the ‘interactions checker’ section on this website, the generic names of the drugs were added one by one to the ‘add’ section, the ‘check for interaction’ option was selected, and the drug interactions were classified into three classes, namely minor, intermediate, and major [ 10 ].

The common dental drugs selected for this study included the antibiotics (penicillin, metronidazole, and amoxicillin), local anesthetics (lidocaine, epinephrine, and prilocaine), analgesics (gelofen, ibuprofen, and acetaminophen), antivirals (acyclovir), antifungals (fluconazole), and oral sedatives (diazepam) [ 12 - 15 ]. As for the patients that were unable to answer the questions, their companions were asked to answer the questions. 

The analysis of the resulting data was performed in SPSS version 25 and the independent sample t test, chi-square test; ANOVA, Spearman’s correlation test, Kruskal-Wallis test, and Mann-Whitney test were carried out to compare the variables. Besides, p< 0.05 was considered the significance level.

## Results

Of the 150 patients participating in this study, 78 (52%) were female and the average age was 71.27±6.32 years. Most patients (39.3%) had a high school diploma. Table 1 presents the demographic properties of the study population. The average age of the female and male participants was 71.3±6.78 and 71.18±5.83 years, respectively. The results of the two-sample independent sample t-test revealed the lack of a significant difference between the female and male patients with regard to age (p= 0.86) (Table 1). The frequency distribution of patients with systemic diseases is shown in Table 2. The most common diseases were hypertension (57.3%), heart diseases (42.0%) and diabetes mellitus (40.7%) in the order mentioned (Table 2). Our analysis of the drugs used by the elderly in this study showed that a total of 95 types of drugs were used by this population. Table 3 lists the most commonly used drugs from each drug family (Table 3). The drug interactions between the common dental drugs including penicillin, fluconazole, lidocaine, ibuprofen, amoxicillin, metronidazole, prilocaine, acetaminophen, acyclovir, and diazepam and 95 drugs used by the elderly were analyzed. A total of 212 DDIs were found. Of these 212 interactions, 46(21.7%) were minor, 145 (68.3%) were moderate and 21(9.9%) were major interactions.

**Table 1:** The demographic properties of the study population

Variable	Frequency	Percent
Sex		
Male	78	52
Female	72	48
Education		
Illiterate	10	6.7
Primary	17	11.3
High school	33	20
Diploma	59	39.3
Above diploma	15	10
Bachelor	13	8.7
Masters	1	0.7
PhD	1	0.7

**Table 2:** The frequency distribution of patients with systemic diseases

Systemic diseases	N	%	Systemic	diseases	N	%
Hypertension	86	57.3	Hyperuricemia	2	1.3
Heart Diseases	63	42.0	Sleep disorders	2	1.3
Diabetes mellitus	61	40.7	Sleep disorders	2	1.3
Hyperlipidemia	61	40.7	Breast Cancer	1	0.7
Neurological Disorders	22	14.7	Allergy	1	0.7
Gastrointestinal Disease	18	12.0	Blood Diseases	1	0.7
Hypothyroidism	10	6.7	Anemia	1	0.7
Respiratory and Pulmonary Diseases	8	5.4	Liver Cyst	1	0.7
Benign Prostate Enlargement	8	5.4	Light Brain Stroke	1	0.7
Kidney Diseases	7	4.6	Vitamin D Deficiency	1	0.7
Rheumatoid arthritis	6	4.0	Breast Cancer	1	0.7
Osteoporosis	5	3.3	Sleep disorders	2	1.3
Lumbar Disk Disease	5	3.3	Breast Cancer	1	0.7
Mental Disorders	5	3.3	Allergy	1	0.7
Osteoarthritis	3	2.0			
Migraine	3	2			

**Table 3:** The most commonly used drugs from each drug family

Drug Family	Drug Name	Frequency	Percent
Antihypertensive	Losartan	54	36
Antiglycemic	Metformin	45	30
Antihyperlipidemic	Atorvastatin	47	31.3
Heart Disease Drugs	Aspirin	33	22
Antihypothyroid	Levothyroxine	10	6.7
Antidepressant	Fluoxetine	1	0.7
Gastrointestinal Drugs	Omeprazole	3	2
Kidney Failure Drugs	Furosemide	6	4
Anti-Alzheimer	Yasnal	3	2
Disease-modifying antirheumatic drugs (DMARDs)	Methotrexate	2	1.3
Osteoporosis Drugs	Alendronate	2	1.3
Antiasthmatic	Salbutamol	3	2
Anti-epileptic	Gabapentin	6	4
Antinausea	Dimenhydrinate	2	1.3
Drugs for reducing uric acid	Allopurinol	2	1.3
Anti-Parkinson	Levodopa	1	0.7
Sedative	Chlordiazpoxide	4	2.7
Sleep disorders Drugs	Alprazolam	3	2
Anticancer	Aromasine	1	0.7
Anemia Drugs	Folic Acid	1	0.7
Multiple Sclerosis Drugs	Dimethyl fumarate	1	0.7
Muscle relaxer	Tizanidine	1	0.7
Analgesic	Celecoxib	3	2
Anthelmintic	Albendazole	1	0.7

**Figure 1:**
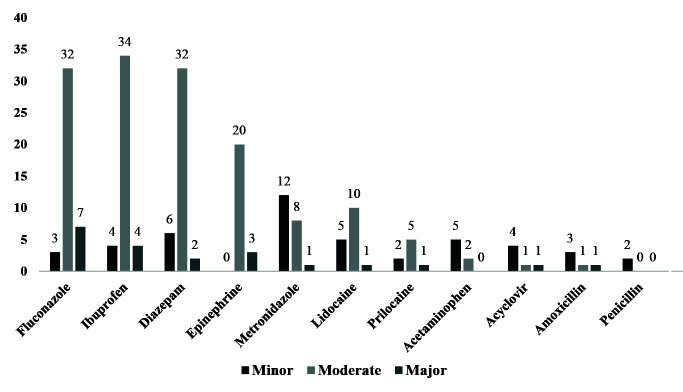
The number and type of the drug interactions found between the drugs used by the elderly in this study and the common dental drugs

The lowest number of interactions between the commonly prescribed dental drugs was associated with penicillin and the highest number of interactions was associated with fluconazole and ibuprofen (which inter acted with 42 of the 95 studied drugs) (Figure 1). It is also worth noting that the moderate interactions were the most prevalent interactions. Of the 95 studied drugs, only two drugs namely fluticasone/salmetrol and symbicort (both antiasthmatic drugs) had interactions with all of the 11 common dental drugs, and the interactions were mainly minor (Table 4). 

**Table 4:** Drugs with the highest number of interactions from each drug category with the common dental drugs

Disease	Drug	Amox	Pen	Met	Pril	Epi	Ibu	Lid	Ace	Flu	Acy	Dia
Antihypertensive	losartan						mo			mo		mo
Antiglycemic	Glibenclamide					mo	mo			ma		
Antihyperlipidemic	Rosuvastatin Atorvastatin			mo						mi		mi
				mo						ma		ma
Heart Disease Drugs	Warfarin	mo		ma	mo		ma		mi	ma		
(DMARDs)	Methotrexate	ma	ma				ma		mo	mo	mo	
Gastrointestinal Disease Drugs	Famotidine Omepraole			mi			mi	mi	mi	mo	mo	mo
										mo		
Neurological and Depression	Sertraline			mi			mo		mo	mo	mo	mi
Osteoporosis	Alendronate						mo					
Analgesia	Celecoxib Diclofenac			mo			mo			mo	mo	
							mo			mo		
+Hypothyroidism	Levothyroxine					mo						
High Uric Acid	Allopurinol	mi										
Prostate	Tamsulosin									mo	mo	mo
Kidney failure	Furosemide						mo					mo
Asthma	Fluticasone/Salmetrol	mi	mi	mi	mi	mi	mo	mi	mi	mi	mo	mi	mo
	Symbicort spray	mi	mi	mi	mi	mo	mo	mi	mi	mo	mi	mi
Alzheimer	Yasnal			mo			mi			mi	mi	
Multiple Sclerosis	Tizanidine			mi						mo	ma	mo
Sleep Disorders	Chlordiazpoxide				mo			mo			mo	mo
	Alprazolam				mo			mo		ma	ma	
Epilepsy	Carbamazepine			mo				mi	mo	mo	mo	mo
Liver Cyst	Albendazole							mi				
Parkinson	Levodopa			mo		mo						mo

DMARDs =Disease-modifying antirheumatic drugs, mo=moderate, ma=major, mi=minor

Amox=Amoxicillin, Pen=Penicillin, Met= Metronidazole, Pril= Prilocaine, Epi=Epinephrine

Ibu= Ibuprofen, Lid= Lidocaine, Ace= Acetaminophen, Flu+ Fluconazole, Acy=Acyclovir, Dia=Diazepam

The results of the Mann Whitney test also showed that there was not a significant difference between the two male and female patient groups with regard to the number of interactions (p=.31). The results of the Kruskal-Wallis test also showed the lack of a statistically significant difference between the patients with different education levels with regard to the number of drug interactions.

The results of Spearman’s correlation test revealed a statistically significant and direct relationship between the number of drug interactions and the number of drugs used by the patients (p= 0.0001, Correlation Coefficient =.87).

## Discussion

The likelihood of incidence of chronic diseases increases with age. Due to the presence of these diseases, the elderly form the largest group of drug users in different societies. The increase in the population age and life expectancy results in an increase in the demand for health care including the use of drugs [ 16 - 17 ]. The excessive intake of drugs may increase the risk of drug interactions, drug errors, side effects, hospitalization frequency, and treatment costs. Moreover, drug interactions are linked to an increase in hospitalization and mortality rates. The results of this study revealed that the total number of interactions between the 95 drugs used and the 11 common dental drugs was 212. Most of these interactions were moderate (145 cases) and minor (46 cases), and the major interactions had the smallest share (21 cases).

In the study by Torkashvand *et al.* [ 17 ] on cardiovascular cases, the total number of interactions was 4318: 62% moderate interactions and 37% major interactions. Alkan *et al.* [ 18 ] also reported that approximately one-third of older cancer patients were exposed to major drug interactions. In the study by Murtaza *et al.* [ 19 ], of the 5019 drug interactions identified in the heart patients, 55% were moderate and 45% were major interactions. Previous studies also suggest that moderate interactions are more prevalent than major interactions [ 17 - 19 ]. In the study by Mousavi *et al.* [ 20 ] on 1260 prescriptions, there was at least one interaction in 339 prescriptions (26.9%) and 751 interactions were found in the prescriptions. The major and moderate drug interactions were reported in 7.3% and 75% of the cases, respectively [ 20 ]. In the present study, most of the interactions were moderate. The difference in the prevalence of drug interactions in different studies could be attributed to the software used for identifying the drug interactions, the different designs of the studies, and the differences in the study populations. 

The most common interactions among the common dental drugs were observed between fluconazole and ibuprofen. In a study by Rashidi *et al.* [ 21 ] 1800 prescriptions with drug interactions were studied. Of all the drug interactions identified, 15.6%, 32.6%, and 41.8% were major, moderate, and minor drug interactions, respectively. They showed that Atenolol had the highest number of drug interactions of the moderate type with none steroidal anti-inflammatory drugs with a share of 11.9%, while the interactions of penicillin with tetracycline and doxycycline were major with a share of 5.5%. Besides, aspirin had the highest number of interactions of the minor type with diphenhydramine and expectorant with a share of 4%.In the present study, most interactions occurred between the antiasthmatic drugs fluticasone/salmetrol and symbicort, which interacted with all 11 common dental drugs. These interactions were mainly minor. According to Merel *et al.* [ 9 ], the most important drug interactions were the interactions with some drug groups including the statins, calcium channel blockers, warfarin, and factor X inhibitors. Pagno *et al.* [ 4 ] reported that 86.3% of the elderly used drugs, and 39.4% of them were exposed to polypharmacy, while 52.2% were exposed to potential drug interactions. Moreover, the drug interactions were mainly caused by enalapril and metformin.

Schneider *et al.* [ 22 ] reported that antiplatelet drugs and none steroidal anti-inflammatory drugs had the highest DDIs, and none steroidal anti-inflammatory drugs had the highest rate of contraindications among all drug categories. In any case, the present study analyzed the drug interactions with dental drugs and its results were, evidently, different from the other studies with regard to the drug types. This study also revealed that the elderly who participated in this study mainly suffered from hypertension (57.3%), diabetes mellitus (40.7%), and hyperlipidemia (39.3%) and the most commonly used drugs were Valsartan (36%), Atorvastatin (31.3%), and Metformin (30%). In the study by Mizokami *et al.* [ 23 ] on participants aged 65 years and higher, the most common diseases were hypertension, hyperlipidemia, and gastric ulcers. These researchers concluded that physicians must carefully take into account the type of the chronic disease in assessing the risk of polypharmacy. Elderly patients with multiple diseases may be more exposed to polypharmacy.

Studies have indicated that many factors such as the increased risk of multiple acute and chronic diseases, increased access to various over-the-counter drugs, changes in patient expectations, and changes in the health care service systems are involved in the increased use of drugs by the elderly [ 22 - 23 ]. Newer, better, and more powerful drugs and medicines are produced every day. Since the elderly are expected to develop diseases and make physical complaints more than the youth, the use of drugs increases drastically among the elderly [ 22 - 23 ]. All of the elderly, especially people over the age of 85, use between five and eight types of drugs every day on average [ 24 ]. In the present study, polypharmacy was observed in 94% of the cases. In the study by Ahmadi and Mahmoodi [ 25 ], the rate of polypharmacy was 36.9% and in the study by Yavari *et al.* [ 26 ] in the Kahrizak nursing home, the rate of polypharmacy was 52.3%. In the study by Haider *et al.* [ 27 ] in Sweden, the rate of polypharmacy was 42.2%. The differences in the findings could be attributed to the differences in the demographic properties, differences in rules and regulations governing drug prescription, and the prevalence of different diseases in the study populations.

Healthcare personnel, especially physicians and nurses, have an important role in the rational prescription and correct use of drugs. Physicians must try to prescribe the minimum number of drugs to treat a patient to prevent the elderly from making mistakes in taking their drugs and reduce the drug side effects [ 28 ]. By educating the elderly or their caregivers, nurses can play a major role in preventing the risks of polypharmacy. Nurses can also explain the effect of the prescribed drugs, the method, time, and amount of drug use, the storage conditions, and the drug and food interactions for each drug in simple and comprehensible terms before the patient leaves the hospital or the clinic [ 28 ].

The results of the present study revealed the lack of a significant difference between the two female and male groups with regard to the number of drug interactions. This finding is similar to the results of the other studies including the studies by Koh *et al.* [ 29 ] and Mousavi *et al.* [ 20 ]. However, the study by Torkashvand *et al.* [ 17 ] revealed that the prevalence of drug interactions was higher in women than that in men.

Furthermore, Castioni *et al.* [ 30 ] reported that polypharmacy is not linked to gender, but the elderly, obese individuals, undereducated individuals and smokers are significantly more prone to polypharmacy. In any case, our study did not show a relationship between the education level and drug interactions. The difference between the results of different studies can be attributed to the use of different study populations. The present study suggests that the development of several diseases is the main determinant in the use of many drugs and a subsequent increase in drug interactions in the elderly.

In the present study, there was a significant direct relationship between the number of drug interactions and the number of drugs used. Similar to the present study, many studies such as the studies by Sánchez-Fidalgo *et al.* [ 31 ], George *et al.* [ 32 ], and Mousavi *et al.* [ 20 ] introduced an increase in the number of drugs as one of the most important factors involved in the development of potential drug interactions. Moreover, as the number of drugs rises, the interaction severity grows. The study by George *et al.* [ 32 ] indicated that there is a significant association between the incidence of the DDIs and three or more other diagnosed diseases. The adverse drug interactions also have a significant relationship with three or more chronic diseases, prescription of over 10 drugs and more than seven days of hospitalization [ 32 ]. 

Due to the structural changes in the mouth in the course of aging in the elderly, the rate of their referral to dental centers increases. Meanwhile, the prescription of several oral and dental drugs may be needed. As mentioned, these patients are treated with various drugs because of having multiple diseases. Therefore, dentists must be informed of the drugs their patients are taking and must examine the drug interactions between the patients’ drugs and the drugs they prescribe to prevent the side effects of drug interactions. This is because when more than one type of drug is used in the treatment regimen of these patients, the likelihood of drug interactions and the subsequent complications resulting from these interactions increases. The treatment group must identify these interactions and then evaluate and analyze the resulting information to prescribe an effective treatment regimen with minimum side effects and interactions for the patients. Based on the results of this study, which indicated the high rate of drug interactions, a more powerful monitoring system has to be designed for reducing the rate of drug interactions in these people. As a limitation of this study, it was more difficult to obtain information and interview older patients than the younger patients, and some elderly patients were unable to answer the questions. Moreover, the likelihood of memory errors was another factor in these patients and thus in these cases, their families were asked to answer the questions.

## Conclusion

The results of this study mirrored the high percentage of drug interactions among the patients, revealing the significant relationship between polypharmacy and drug interactions in the elderly patients. Therefore, identifying the DDIs and analyzing the identified relationships with the aim of reducing the drug side effects are useful for increasing patients’ life expectancy and reducing the treatment costs.

## Acknowledgement

This study supported by Oral and Dental Diseases Research Center, Kerman University of Medical Sciences, Kerman, Iran.

## Conflict of Interest

The authors declare that they have no conflict of interest.
